# Bis(*N*-butyl-*N*-ethyl­dithio­carbamato-κ^2^ *S*,*S*′)nickel(II)

**DOI:** 10.1107/S1600536810006677

**Published:** 2010-02-27

**Authors:** Wan Nur Shazwani Wan Juhari, Ibrahim Baba, Yang Farina, Seik Weng Ng

**Affiliations:** a School of Chemical Sciences, Universiti Kebangbaan Malaysia, 43600 Bangi, Malaysia; b Department of Chemistry, University of Malaya, 50603 Kuala Lumpur, Malaysia

## Abstract

The dithio­carbamate anions in the title compound, [Ni(C_7_H_14_NS_2_)_2_], chelate to the Ni^II^ atom, which is four-coordinate in a square-planar geometry. The Ni^II^ atom lies on a center of inversion.

## Related literature

For nickel bis­(diethyl­dithio­carbamate) and nickel bis­(di­butyl­dithio­carbamate), see: Bonamico *et al.* (1965 ▶); Khan *et al.* (1987 ▶); Lokaj *et al.* (1984 ▶).
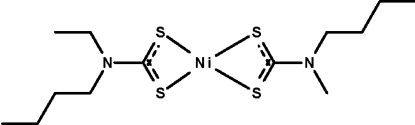


## Experimental

### 

#### Crystal data

[Ni(C_7_H_14_NS_2_)_2_]
*M* *_r_* = 411.33
Monoclinic, 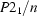

*a* = 8.5641 (9) Å
*b* = 8.6316 (9) Å
*c* = 13.6047 (14) Å
β = 94.753 (2)°
*V* = 1002.23 (18) Å^3^
*Z* = 2
Mo *K*α radiation
μ = 1.38 mm^−1^
*T* = 293 K
0.25 × 0.25 × 0.05 mm

#### Data collection

Bruker SMART APEX diffractometer
Absorption correction: multi-scan (*SADABS*; Sheldrick, 1996 ▶) *T*_min_ = 0.724, *T*_max_ = 0.934
9338 measured reflections
2295 independent reflections
1628 reflections with *I* > 2σ(*I*)
*R*_int_ = 0.029

#### Refinement

*R*[*F* ^2^ > 2σ(*F* ^2^)] = 0.034
*wR*(*F* ^2^) = 0.098
*S* = 1.03
2295 reflections
99 parameters
6 restraints
H-atom parameters constrained
Δρ_max_ = 0.38 e Å^−3^
Δρ_min_ = −0.21 e Å^−3^

### 

Data collection: *APEX2* (Bruker, 2009 ▶); cell refinement: *SAINT* (Bruker, 2009 ▶); data reduction: *SAINT*; program(s) used to solve structure: *SHELXS97* (Sheldrick, 2008 ▶); program(s) used to refine structure: *SHELXL97* (Sheldrick, 2008 ▶); molecular graphics: *X-SEED* (Barbour, 2001 ▶); software used to prepare material for publication: *publCIF* (Westrip, 2010 ▶).

## Supplementary Material

Crystal structure: contains datablocks global, I. DOI: 10.1107/S1600536810006677/sj2734sup1.cif

Structure factors: contains datablocks I. DOI: 10.1107/S1600536810006677/sj2734Isup2.hkl

Additional supplementary materials:  crystallographic information; 3D view; checkCIF report

## Figures and Tables

**Fig. 1.**
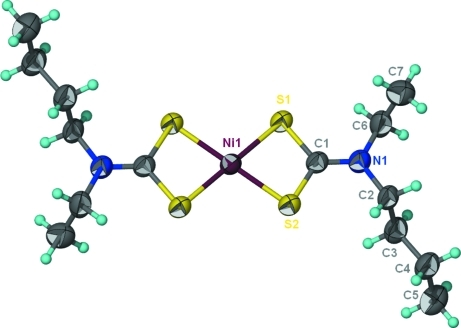
Thermal ellipsoid plot (Barbour, 2001) of Ni(C7H14NS2)2 at the 50% probability level; hydrogen atoms are drawn as spheres of arbitrary radius.

## supplementary crystallographic information

### Experimental

Nickel(II) chloride (10 mmol), butylethylamine (10 mmol) and carbon disulfide (10 mmol) were reacted in ethanol (50 ml) at 277 K to produce a brown solid. The mixture was stirred for an hour. The solid was collected and recrystallized from ethanol.

### Refinement

Carbon-bound H-atoms were placed in calculated positions (C—H 0.96 to 0.97 Å) and were included in the refinement in the riding model approximation, with *U*(H) set to 1.2 to 1.5*U*(C).

### Crystal data

**Table:**

[Ni(C_7_H_14_NS_2_)_2_]	*F*(000) = 436
*M**_r_* = 411.33	*D*_x_ = 1.363 Mg m^−^^3^
Monoclinic, *P*2_1_/*n*	Mo *K*α radiation, λ = 0.71073 Å
Hall symbol: -P 2yn	Cell parameters from 2307 reflections
*a* = 8.5641 (9) Å	θ = 2.4–24.6°
*b* = 8.6316 (9) Å	µ = 1.38 mm^−^^1^
*c* = 13.6047 (14) Å	*T* = 293 K
β = 94.753 (2)°	Plate, brown
*V* = 1002.23 (18) Å^3^	0.25 × 0.25 × 0.05 mm
*Z* = 2	

### Data collection

**Table:**

Bruker SMART APEX diffractometer	2295 independent reflections
Radiation source: fine-focus sealed tube	1628 reflections with *I* > 2σ(*I*)
graphite	*R*_int_ = 0.029
ω scans	θ_max_ = 27.5°, θ_min_ = 2.7°
Absorption correction: multi-scan (*SADABS*; Sheldrick, 1996)	*h* = −10→11
*T*_min_ = 0.724, *T*_max_ = 0.934	*k* = −11→11
9338 measured reflections	*l* = −17→15

### Refinement

**Table:**

Refinement on *F*^2^	Primary atom site location: structure-invariant direct methods
Least-squares matrix: full	Secondary atom site location: difference Fourier map
*R*[*F*^2^ > 2σ(*F*^2^)] = 0.034	Hydrogen site location: inferred from neighbouring sites
*wR*(*F*^2^) = 0.098	H-atom parameters constrained
*S* = 1.03	*w* = 1/[σ^2^(*F*_o_^2^) + (0.0469*P*)^2^ + 0.1755*P*] where *P* = (*F*_o_^2^ + 2*F*_c_^2^)/3
2295 reflections	(Δ/σ)_max_ = 0.001
99 parameters	Δρ_max_ = 0.38 e Å^−^^3^
6 restraints	Δρ_min_ = −0.21 e Å^−^^3^

### Fractional atomic coordinates and isotropic or equivalent isotropic displacement parameters (Å^2^)

**Table:**

	*x*	*y*	*z*	*U*_iso_*/*U*_eq_	
Ni1	0.5000	0.5000	0.5000	0.05331 (16)	
S1	0.75597 (8)	0.48176 (8)	0.53089 (5)	0.0629 (2)	
S2	0.52687 (8)	0.56847 (9)	0.65651 (5)	0.0631 (2)	
N1	0.8339 (3)	0.5601 (3)	0.72015 (17)	0.0647 (6)	
C1	0.7236 (3)	0.5403 (3)	0.64766 (19)	0.0564 (6)	
C2	0.7964 (3)	0.6124 (3)	0.81795 (18)	0.0658 (7)	
H2A	0.7008	0.6732	0.8111	0.079*	
H2B	0.8799	0.6791	0.8456	0.079*	
C3	0.7752 (4)	0.4796 (3)	0.8887 (2)	0.0746 (8)	
H3A	0.6875	0.4163	0.8630	0.090*	
H3B	0.8684	0.4154	0.8928	0.090*	
C4	0.7460 (4)	0.5353 (4)	0.9904 (2)	0.0836 (9)	
H4A	0.6554	0.6032	0.9856	0.100*	
H4B	0.8355	0.5955	1.0167	0.100*	
C5	0.7180 (4)	0.4044 (4)	1.0615 (2)	0.1014 (11)	
H5A	0.6962	0.4470	1.1241	0.152*	
H5B	0.8098	0.3403	1.0699	0.152*	
H5C	0.6305	0.3433	1.0356	0.152*	
C6	1.0008 (4)	0.5276 (4)	0.7074 (2)	0.0805 (9)	
H6A	1.0079	0.4559	0.6530	0.097*	
H6B	1.0490	0.4791	0.7667	0.097*	
C7	1.0871 (4)	0.6734 (4)	0.6871 (2)	0.0951 (10)	
H7A	1.1957	0.6500	0.6818	0.143*	
H7B	1.0780	0.7453	0.7401	0.143*	
H7C	1.0432	0.7183	0.6265	0.143*	

### Atomic displacement parameters (Å^2^)

**Table:**

	*U*^11^	*U*^22^	*U*^33^	*U*^12^	*U*^13^	*U*^23^
Ni1	0.0586 (3)	0.0553 (3)	0.0464 (3)	−0.0031 (2)	0.0063 (2)	−0.00089 (19)
S1	0.0628 (4)	0.0763 (4)	0.0504 (4)	0.0013 (3)	0.0095 (3)	−0.0089 (3)
S2	0.0589 (4)	0.0785 (5)	0.0526 (4)	0.0006 (3)	0.0090 (3)	−0.0081 (3)
N1	0.0596 (13)	0.0780 (14)	0.0572 (13)	0.0057 (11)	0.0077 (11)	−0.0171 (12)
C1	0.0629 (16)	0.0556 (14)	0.0513 (15)	−0.0005 (11)	0.0083 (12)	−0.0052 (11)
C2	0.0670 (17)	0.0758 (17)	0.0542 (16)	0.0016 (13)	0.0023 (13)	−0.0198 (13)
C3	0.080 (2)	0.080 (2)	0.0629 (18)	0.0053 (14)	0.0010 (15)	−0.0130 (15)
C4	0.093 (2)	0.096 (2)	0.0621 (19)	0.0055 (17)	0.0047 (17)	−0.0136 (17)
C5	0.118 (3)	0.111 (3)	0.074 (2)	0.001 (2)	0.002 (2)	0.000 (2)
C6	0.0653 (18)	0.109 (3)	0.0660 (19)	0.0082 (17)	−0.0034 (15)	−0.0236 (17)
C7	0.076 (2)	0.125 (3)	0.086 (2)	0.008 (2)	0.0171 (17)	−0.006 (2)

### Geometric parameters (Å, °)

**Table:**

Ni1—S1^i^	2.2032 (8)	C3—H3B	0.9700
Ni1—S1	2.2032 (8)	C4—C5	1.519 (4)
Ni1—S2	2.2034 (7)	C4—H4A	0.9700
Ni1—S2^i^	2.2034 (7)	C4—H4B	0.9700
S1—C1	1.712 (3)	C5—H5A	0.9600
S2—C1	1.716 (3)	C5—H5B	0.9600
N1—C1	1.319 (3)	C5—H5C	0.9600
N1—C2	1.466 (3)	C6—C7	1.497 (4)
N1—C6	1.481 (4)	C6—H6A	0.9700
C2—C3	1.517 (4)	C6—H6B	0.9700
C2—H2A	0.9700	C7—H7A	0.9600
C2—H2B	0.9700	C7—H7B	0.9600
C3—C4	1.505 (4)	C7—H7C	0.9600
C3—H3A	0.9700		
			
S1^i^—Ni1—S1	180.0	H3A—C3—H3B	107.9
S1^i^—Ni1—S2	100.82 (2)	C3—C4—C5	113.3 (3)
S1—Ni1—S2	79.18 (2)	C3—C4—H4A	108.9
S1^i^—Ni1—S2^i^	79.18 (2)	C5—C4—H4A	108.9
S1—Ni1—S2^i^	100.82 (2)	C3—C4—H4B	108.9
S2—Ni1—S2^i^	180.0	C5—C4—H4B	108.9
C1—S1—Ni1	85.45 (10)	H4A—C4—H4B	107.7
C1—S2—Ni1	85.34 (9)	C4—C5—H5A	109.5
C1—N1—C2	121.4 (2)	C4—C5—H5B	109.5
C1—N1—C6	121.7 (2)	H5A—C5—H5B	109.5
C2—N1—C6	116.8 (2)	C4—C5—H5C	109.5
N1—C1—S1	124.8 (2)	H5A—C5—H5C	109.5
N1—C1—S2	125.1 (2)	H5B—C5—H5C	109.5
S1—C1—S2	110.04 (16)	N1—C6—C7	111.0 (3)
N1—C2—C3	113.0 (2)	N1—C6—H6A	109.4
N1—C2—H2A	109.0	C7—C6—H6A	109.4
C3—C2—H2A	109.0	N1—C6—H6B	109.4
N1—C2—H2B	109.0	C7—C6—H6B	109.4
C3—C2—H2B	109.0	H6A—C6—H6B	108.0
H2A—C2—H2B	107.8	C6—C7—H7A	109.5
C4—C3—C2	112.3 (2)	C6—C7—H7B	109.5
C4—C3—H3A	109.1	H7A—C7—H7B	109.5
C2—C3—H3A	109.1	C6—C7—H7C	109.5
C4—C3—H3B	109.1	H7A—C7—H7C	109.5
C2—C3—H3B	109.1	H7B—C7—H7C	109.5
			
S2—Ni1—S1—C1	−0.24 (9)	Ni1—S1—C1—S2	0.32 (12)
S2^i^—Ni1—S1—C1	179.76 (9)	Ni1—S2—C1—N1	180.0 (2)
S1^i^—Ni1—S2—C1	−179.76 (9)	Ni1—S2—C1—S1	−0.32 (12)
S1—Ni1—S2—C1	0.24 (9)	C1—N1—C2—C3	94.2 (3)
C2—N1—C1—S1	179.3 (2)	C6—N1—C2—C3	−84.6 (3)
C6—N1—C1—S1	−2.0 (4)	N1—C2—C3—C4	176.7 (3)
C2—N1—C1—S2	−1.0 (4)	C2—C3—C4—C5	177.8 (3)
C6—N1—C1—S2	177.6 (2)	C1—N1—C6—C7	98.5 (3)
Ni1—S1—C1—N1	−180.0 (2)	C2—N1—C6—C7	−82.8 (3)

Symmetry codes: (i) −*x*+1, −*y*+1, −*z*+1.
